# Nanostructured Molecular–Network Arsenoselenides from the Border of a Glass-Forming Region: A Disproportionality Analysis Using Complementary Characterization Probes

**DOI:** 10.3390/molecules29163948

**Published:** 2024-08-21

**Authors:** Oleh Shpotyuk, Malgorzata Hyla, Adam Ingram, Yaroslav Shpotyuk, Vitaliy Boyko, Pavlo Demchenko, Renata Wojnarowska-Nowak, Zdenka Lukáčová Bujňáková, Peter Baláž

**Affiliations:** 1 Institute of Physics, Jan Dlugosz University in Częstochowa, 13/15, al. Armii Krajowej, 42-200 Częstochowa, Poland; m.hyla@ujd.edu.pl; 2 O.G. Vlokh Institute of Physical Optics, Ivan Franko National University of Lviv, 23, Dragomanov Str., 79005 Lviv, Ukraine; boykovit@gmail.com; 3 Faculty of Physics, Opole University of Technology, 75, Ozimska Str., 45370 Opole, Poland; a.ingram@po.edu.pl; 4 Department of Sensor and Semiconductor Electronics, Ivan Franko National University of Lviv, 107, Tarnavskoho Str., 79017 Lviv, Ukraine; yashpotyuk@gmail.com; 5 Institute of Physics, University of Rzeszow, 1, Pigonia Str., 35-959 Rzeszow, Poland; 6 Department of Inorganic Chemistry, Ivan Franko National University of Lviv, 6, Kyryla i Mefodiya Str., 79000 Lviv, Ukraine; pavlo.demchenko@lnu.edu.ua; 7 Center for Microelectronics and Nanotechnology, Institute of Materials Engineering, University of Rzeszow, 1, Pigonia Str., 35-959 Rzeszow, Poland; rwojnarowska@ur.edu.pl; 8 Institute of Geotechnics of Slovak Academy of Sciences, 45, Watsonova Str., 04001 Košice, Slovakia; bujnakova@saske.sk (Z.L.B.); balaz@saske.sk (P.B.)

**Keywords:** thioarsenides, arsenoselenides, molecules, network-forming entities, glass-forming region, nanostructurization, nanomilling, X-ray powder diffraction (XRPD), Raman scattering (RS) microscopy, positron annihilation lifetime (PAL), free-volume defects, positronium (Ps)

## Abstract

Binary As_x_Se_100−x_ alloys from the border of a glass-forming region (65 < *x* < 70) subjected to nanomilling in dry and dry–wet modes are characterized by the XRPD, micro-Raman scattering (micro-RS) and revised positron annihilation lifetime (PAL) methods complemented by a disproportionality analysis using the quantum–chemical cluster modeling approach. These alloys are examined with respect to tetra-arsenic biselenide As_4_Se_2_ stoichiometry, realized in glassy g-As_65_Se_35_, glassy–crystalline g/c-As_67_Se_33_ and glassy–crystalline g/c-As_70_Se_30_. From the XRPD results, the number of rhombohedral As and cubic arsenolite As_2_O_3_ phases in As-Se alloys increases after nanomilling, especially in the wet mode realized in a PVP water solution. Nanomilling-driven amorphization and reamorphization transformations in these alloys are identified by an analysis of diffuse peak halos in their XRPD patterning, showing the interplay between the levels of a medium-range structure (disruption of the intermediate-range ordering at the cost of an extended-range one). From the micro-RS spectroscopy results, these alloys are stabilized by molecular thioarsenides As_4_Se_n_ (*n* = 3, 4), regardless of their phase composition, remnants of thioarsenide molecules destructed under nanomilling being reincorporated into a glass network undergoing a polyamorphic transition. From the PAL spectroscopy results, volumetric changes in the wet-milled alloys with respect to the dry-milled ones are identified as resulting from a direct conversion of the bound positron–electron (Ps, positronium) states in the positron traps. Ps-hosting holes in the PVP medium appear instead of positron traps, with ~0.36–0.38 ns lifetimes ascribed to multivacancies in the As-Se matrix. The superposition of PAL spectrum peaks and tails for pelletized PVP, unmilled, dry-milled, and dry–wet-milled As-Se samples shows a spectacular smoothly decaying trend. The microstructure scenarios of the spontaneous (under quenching) and activated (under nanomilling) decomposition of principal network clusters in As_4_Se_2_-bearing arsenoselenides are recognized. Over-constrained As_6·(2/3)_ ring-like network clusters acting as pre-cursors of the rhombohedral As phase are the main products of this decomposition. Two spontaneous processes for creating thioarsenides with crystalline counterparts explain the location of the glass-forming border in an As-Se system near the As_4_Se_2_ composition, while an activated decomposition process for creating layered As_2_Se_3_ structures is responsible for the nanomilling-driven molecular-to-network transition.

## 1. Introduction

The binary arsenoselenides of an As_x_Se_100−x_ cut-section comprise an important family of substances with saturated covalent bonding [1,2,3,4], where glass-like specimens can easily be obtained via high-entropy melting by quenching (melt-quenching, MQ) within an unprecedentedly wide compositional domain 0 ≤ *x* < ~70 corresponding to coordination numbers (CN) 2.0 < CN < ~2.70 [1,2]. Because of their vitreous state, these materials represent promising candidates for multifunctional applications in electronics, optoelectronics, IR optics, chemical and biochemical sensing, etc. [4,5,6]. In the early 2010s, the achieved progress in such implementation spheres was so impressive that a new branch of IR photonics, nominated Chalcogenide Photonics, appeared [7,8].

Nowadays [9,10,11], it is obvious that the most important functionality of arsenoselenides is governed by the specificity of their vitreous state, which can be modified either intrinsically, owing to the optimized technology of MQ, or extrinsically, owing to the post-technological influence on MQ-derived alloys. As for the latter, essential progress has been achieved in the most recent decade on so-called nanostructurization routines like high-energy mechanical milling (MM), also termed nanomilling [12], applied to transfer these substances from the macro- to nanoscopic scales, exploring the unique possibilities of their newly attained nanofunctionality [13,14,15,16,17]. From this point, the As-bearing arsenoselenides beyond stoichiometric arsenic triselenide As_2_Se_3_ (*x* > 40), which possess glass-like structures due to the thioarsenide As_4_Se_n_ molecules stabilized in the network of remaining As-Se bonds, are especially attractive [1,2,3]. In this research, the thioarsenide nomenclature As_4_X_n_ recommended for As-rich over-stoichiometric sulphides (X = S) under an analysis of the molecular-packing conformations by Bonazzi and Bindi [18] and the electron density by Gibbs et al. [19] is used for As_x_Se_100−x_ alloys. A milling-driven escape towards an optimized nanostructured state occurs in these substances via transitions between the amorphous states, referred to as polyamorphic or reamorphization transitions, similar to the characteristics of chemically stoichiometric arsenic triselenide As_2_Se_3_ (corresponding to *x* = 40 in As_x_Se_100−x_, CN = 2.40) and under-stoichiometric Se-rich glassy alloys (0 < *x* < 40; 2.0 < CN < 2.40) [20,21]. In contrast, in compositional domains where some molecular species can be stabilized in a glass-forming network, transitions between different crystalline and amorphous states (referred to as polymorphic and polyamorphic phase transitions) can be activated by nanomilling in a dry mode, like in Se-rich As_x_Se_100−x_ glassy alloys (*x* < 40), approaching ‘pure’ Se (0 ≤ *x* < ~10–12) [22] or As-rich alloys (*x* > 40) of arsenic monoselenide, g-AsSe (also referred to as tetra-arsenic tetraselenide, g-As_4_Se_4_) [23]. Although such transitions have a higher content of side-oxidized derivatives, they can be activated in alloys subjected to nanomilling in a wet and/or combined dry–wet mode, typically realized in an environment with some surfactants, such as the PVP (polyvinylpyrrolidone) water solution [24,25,26].

Specifically, it is expected that both the polymorphic and polyamorphic phase transitions can be activated in the MQ-derived As_x_Se_100−x_ alloys at the border of the glass-forming region ~(65 < *x* < 70) enriched in glassy and glassy–crystalline species undergoing nanostructurization by means of MM, with this process being dependent on the MM mode (dry or wet). There are no crystalline counterparts to molecular thioarsenides at the border of the glass-forming region in an As-Se system such as tetra-arsenic biselenide As_4_Se_2_ (corresponding to CN = 2.67, which is just at the edge of the glass-forming ability in this system [1,2]), while some thioarsenide molecules in a nearby region (such as tetra-arsenic triselenide As_4_Se_3_ with CN = 2.67, or tetra-arsenic monoselenide As_4_Se with CN = 2.80) are still expected under grinding in a high-energy ball mill, serving as a source of possible network-forming derivatives contributing to the final state of these nanostructured substances.

The scope of this work is comprehensive research on the As_x_Se_100−x_ alloys from the border of the glass-forming region (2.65 < CN < 2.70) subjected to nanomilling in dry- and combined dry–wet modes, using multi-experimental characterization probes like X-ray powder diffraction (XRPD) analysis in terms of a modified microcrystalline model [20,21,22,23], micro-Raman scattering (micro-RS) spectroscopy [27] and revised positron annihilation lifetime (PAL) analysis within the *Positronics* approach [28,29], complemented with molecular–network disproportionality analysis in the As-Se system employing ab initio quantum–chemical cluster-modeling algorithm CINCA like in [22,23]. In the current research, the compositional domain of interest in the As-Se system, which will be shifted towards the border of the glass-forming region, exemplified by arsenoselenide alloys deviated around tetra-arsenic biselenide As_4_Se_2_ *thioarsenide* stoichiometry, these being under-stoichiometric glassy g-As_65_Se_35_ (CN = 2.65), stoichiometric glassy-crystalline g/c-As_67_Se_33_ (*viz*. g/c-As_4_Se_2_, CN = 2.67) and over-stochiometric glassy-crystalline g/c-As_70_Se_30_ (CN = 2.70).

## 2. Results and Discussion

### 2.1. Atomic-Specific Microstructure of the Examined Arsenoselenides

**Figure 1:**
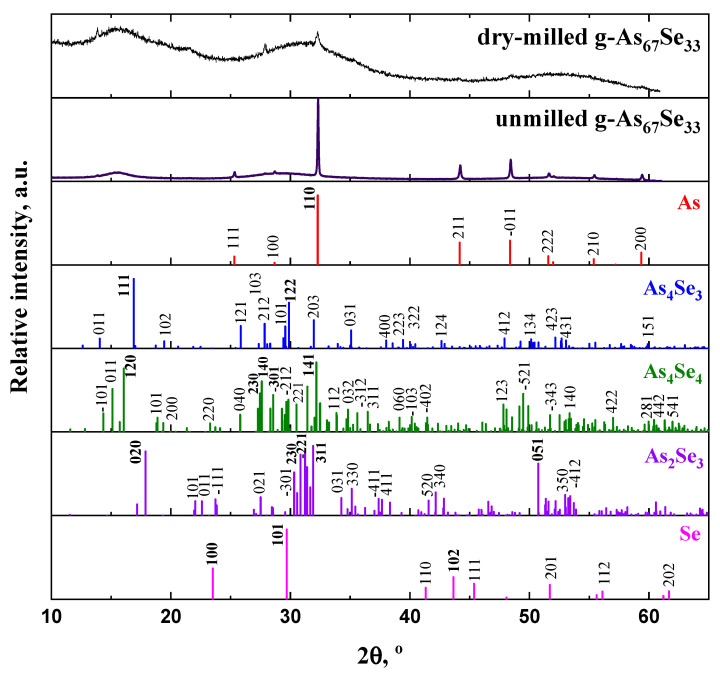
The normalized XRPD patterns of MQ-derived g/c-As_67_Se_33_ in unmilled and dry-milled state showing three principle diffuse peak halos responsible for the FSDP (~15–25°2*θ*), SSDP (~28–33°2*θ*) and TDP (~50–60°2*θ*). The Bragg-diffraction reflexes of crystalline counterparts are reproduced (from the top to the bottom) in a sequence: rhombohedral (grey) As (JCPDS No. 72–1048) [30,31], orthorhombic As_4_Se_3_ (JCPDS No. 04–4979) [32], monoclinic As_4_Se_4_ (JCPDS No. 71–0388) [33,34], monoclinic As_2_Se_3_ (JCPDS No. 65–2365) [35,36] and trigonal Se (JCPDS No. 73–0465) [37].

**Figure 2:**
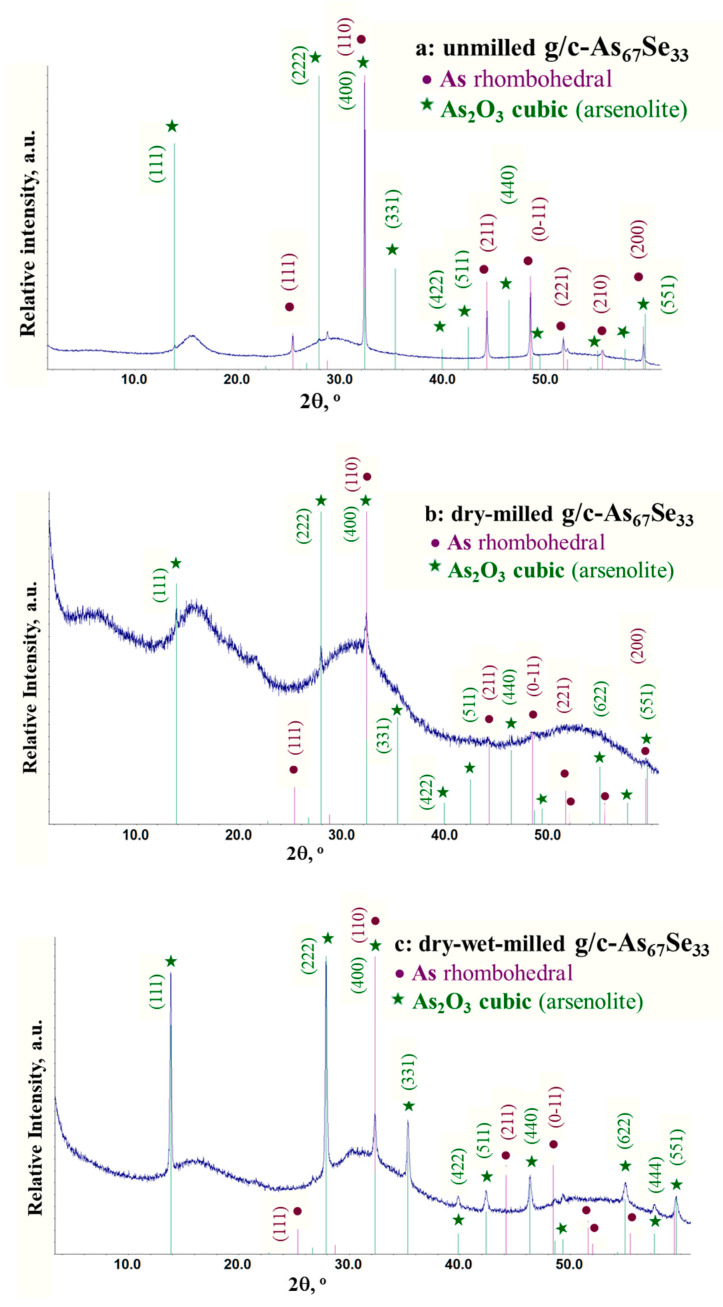
The normalized XRPD patterns of MQ-derived g/c-As_67_Se_33_ before (**a**) and after nanomilling in dry (**b**) and combined dry–wet (**c**) mode showing three principal diffuse peak halos in comparison with the Bragg-diffraction reflexes from rhombohedral As (JCPDS No. 72–1048) [30,31] and cubic arsenolite As_2_O_3_ phase (JCPDS No. 36–1490) [38].

The normalized XRPD profiles of unmilled and dry-milled g/c-As_67_Se_33_ alloy (CN = 2.67) strictly corresponding to tetra-arsenic biselenide (As_4_Se_2_) thioarsenide stoichiometry along with the Bragg diffraction reflexes from possible As-Se crystalline counterparts [30,31,32,33,34,35,36,37] are reproduced in Figure 1. These XRPD patterns demonstrate a so-called three-peak amorphous structure of this substance composed of three principal diffuse peak halos responsible for the FSDP (the first sharp diffraction peak) at ~15–25°2*θ*, SSDP (the second sharp diffraction peak) at ~28–33°2*θ* and TDP (the third diffraction peak) at ~50–60^o^2*θ*, superimposed with sharp-broadened Bragg diffraction reflexes from nanocrystalline inclusions of elemental As [30,31] and its oxide As_2_O_3_ [38]. In part, analysis of the XRPD patterns reproduced in Figure 1 and Figure 2 for g/c-As_67_Se_33_ samples of different pre-history confirms that the strongest diffraction lines arise from the planes (110), (0–11) and (211) in rhombohedral As [30,31], and (222), (111), (331), (400) and (440) in cubic arsenolite As_2_O_3_ [38].

In unmilled g/c-As_67_Se_33_ (Figure 2a), the elemental As is dominated over its oxide (As_2_O_3_) due to the sharp ‘nanocrystalline’ reflexes of this phase superimposed on relatively weak ‘amorphous’ halos, the FSDP-related diffuse peak halo being equilibrated by intensity with that responsible for the SSDP at ~28–33°2*θ*, which is characteristic of vitreous selenides [39,40].

In dry-milled g/c-As_67_Se_33_ treated in a protective argon atmosphere (Figure 2b), the diffuse peak halos prevail over broadened ‘nanocrystalline’ reflexes from elemental As and its oxide, confirming that strong amorphous-I-to-amorphous-II (reamorphization) transition in this sample is probably accompanied by crystalline-to-amorphous (amorphization) transition, as this occurs in g-AsSe subjected to high-energy MM in a dry mode [23]. All three principal diffuse peak halos in dry-milled g/c-As_67_Se_33_ fit in a decaying sequence on their intensities like in Sulphur S-bearing glasses [39], meaning that stress generated under grinding does not completely relax over the glass network.

**Figure 3:**
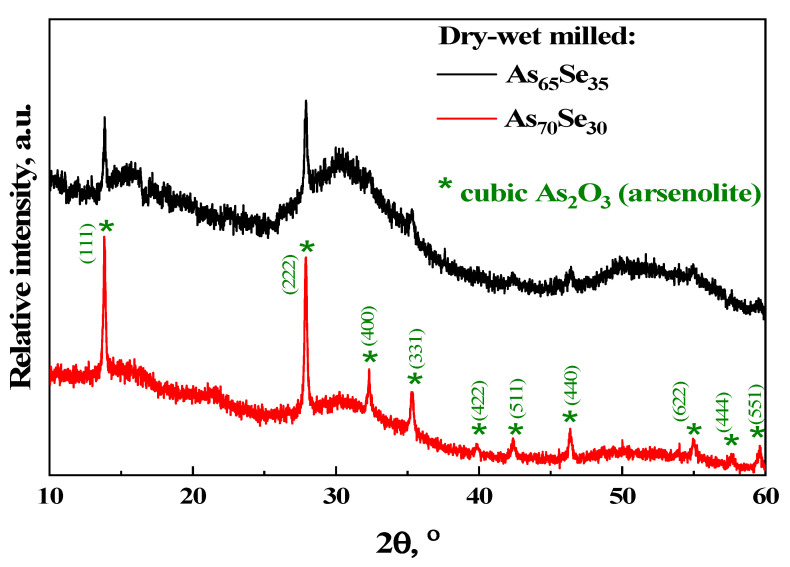
The normalized XRPD patterns of g-As_65_Se_35_ (black curve) and g/c-As_70_Se_30_ (red curve) after nanomilling in dry–wet mode, showing three principal diffuse peak halos corresponding to ‘amorphous’ phase overlapped with sharp broadened Bragg-diffraction reflexes originated from the planes (111) at 13.86°2*θ* (*d* = 6.390Å, *I* = 63%), (222) at 27.90°2*θ* (*d* = 3.195Å, *I* = 100%), (400) at 32.33°2*θ* (*d* = 2.769Å, *I* = 27%), (331) at 35.32°2*θ* (*d* = 2.541 Å, *I* = 38%), (440) at 46.36°2*θ* (*d* = 1.957Å, *I* = 27%) and (551) at 59.59°2*θ* (*d* = 1.551 Å, *I* = 27%) in cubic structure of arsenolite As_2_O_3_ (JCPDS No. 36–1490) [38].

Then, in dry-milled g/c-As_67_Se_33_ additionally treated in a wet mode (that is, in dry–wet-milled samples, see Figure 2c), ‘nanocrystalline’ lines of As_2_O_3_ phase abnormally grow in intensity and become broader in width over those of elemental As, and the arrangement of principal peak halos corresponding to the amorphous phase attains a characteristic irregularity, which dominated the SSDP-related peak halo at ~28–33°2*θ* as a signature of thermodynamically stabilized glassy alloys from the glass-forming region [20,21,22,23]. Therefore, it seems quite reasonable that MM in the PVP water solution promotes further extraction and oxidation of the As phase. In the g-As_65_Se_35_ sample from the edge of the glass-forming region, which was in the vitreous state before and after dry MM [26], nanomilling in a wet mode results in an arsenolite As_2_O_3_ phase without a notable admixture of the unoxidized elemental As phase (Figure 3). In line with our expectation, this oxidation process is enhanced under the transition to glassy-crystalline alloys from the border of the glass-forming region such as g/c-As_70_Se_30_ (see Figure 3). 

The MM-driven amorphization and reamorphization processes in the examined arsenoselenides can be adequately identified due to the analysis of the arrangement of diffuse peak halos in their XRPD patterns reproduced in Figure 1, Figure 2 and Figure 3, the derived FSDP parameters related to intermediate-range ordering in these alloys being summarized in Table 1.

From a detailed inspection of Figure 1, the position of the FSDP-related peak halo in unmilled g/c-As_67_Se_33_ at 15.479°2*θ* (equivalent to characteristic distance in the Bragg diffraction *R*~5.71 Å, or average inter-atomic distance in the Ehrenfest diffraction *d_s_* = 7.02 Å) agrees well with the most intensive diffraction line (*I* = 100%) in orthorhombic As_4_Se_3_ which arise from the (111) plane at ~16.90°2*θ* (equivalent to inter-planar distance *d* = 5.243 Å [32]). One of the strongest (*I* = 91.3%) lines in other molecular thioarsenide (monoclinic As_4_Se_4_ [33,34]) arises from the (120) plane at ~16.07°2*θ* (*d* = 5.512 Å), which is also close to the FSDP position. The other line (*I* = 91.2%) ascribed to the (020) plane in monoclinic As_2_Se_3_ [35,36] is revealed at higher diffraction angles ~17.9°2*θ* (*d* = 4.950 Å). Assuming equal contributions from these inter-planar correlations, the FSDP-related characteristic distance R can be found to approach ~5.64 Å, instead of ~5.71 Å from solely the Bragg diffraction positioning of this diffuse peak halo (see Table 1). So, another input to the FSDP is expected from the Ehrenfest diffraction contributing through inter-atomic and/or inter-molecular correlations, which belong to molecular thioarsenide structures and their network-forming derivatives, with average inter-atomic/molecular distances *d_s_* beyond ~7 Å [23].

In As-rich g-As_x_Se_100−x_ (40 < *x* < 65) [21], remnants of thioarsenide cage-like As_4_Se_n_ molecules (such as As_4_Se_4_, As_4_Se_3_, As_4_) are known to be destroyed under MM and re-incorporated into a glass network resulting in an increased FSDP position Q_1_ and width Δ*Q*_1_. This amorphization trend corresponding to the crystalline-to-amorphous transition causes a fragmentation impact on the correlation length of the FSDP-responsible entities (*L*), facilitating changes when distant inter-atomic correlations between some crystalline planes contributing to the FSDP disappear at a cost of others responsible for the SSDP. The disruption of intermediate-range ordering due to weakening of the FSDP-responsible entities accompanied by an enhancement of extended-range ordering due to fragmentation of the SSDP-responsible entities occurs as interplay between different hierarchical levels of the medium-range structure in As-Se alloys from the glass-forming region subjected to MM [21].

Similar peculiarities are found in the examined glassy and glassy-crystalline As-Se alloys from the border of the glass-forming region. Thus, in dry-milled alloys, the FSDP position Q_1_ slightly shifts in the high-angular side, while the FSDP width ΔQ_1_ obeys a drastic increase, resulting in fragmentation of the correlation length L (see Table 1). These changes prevail in glassy-crystalline substances such as g/c-As_70_Se_30_, confirming that crystalline remainders serve as a possible source for the MM-driven amorphization (crystalline-to-amorphous) transition. As shown in Figure 2, nanomilling in combined dry–wet mode (when the last processing stage is performed in the PVP water solution) increases the breakdown in intermediate-range ordering. Nanomilling in wet mode does not change the principal appearance of diffuse peak halos in the XRPD patterns but leads to drastic broadening in their widths, in favor of enhanced reamorphization in preliminary dry-milled samples.

Thus, remnants of ‘crystalline’ structures in the examined alloys responsible for inter-molecular correlations with inter-centroid distances between thioarsenide cage-like molecules above ~7 Å (which contribute to the XRPD patterning by the Ehrenfest diffraction) are destroyed under MM, while the inter-planar correlations contribute further to these patterns through Bragg diffraction. Because of molecular-to-network reamorphization transformations in the examined As-Se alloys, the broadened and depressed diffuse peak halos become shifted towards higher scattering vectors. Since remnants of thioarsenide As_4_Se_n_ molecular entities destructed under grinding interact with oxygen, especially under MM in the PVP water solution, the arsenolite (As_2_O_3_) phase is formed and stabilized in these alloys subjected to nanomilling in combined dry–wet mode.

**Figure 4:**
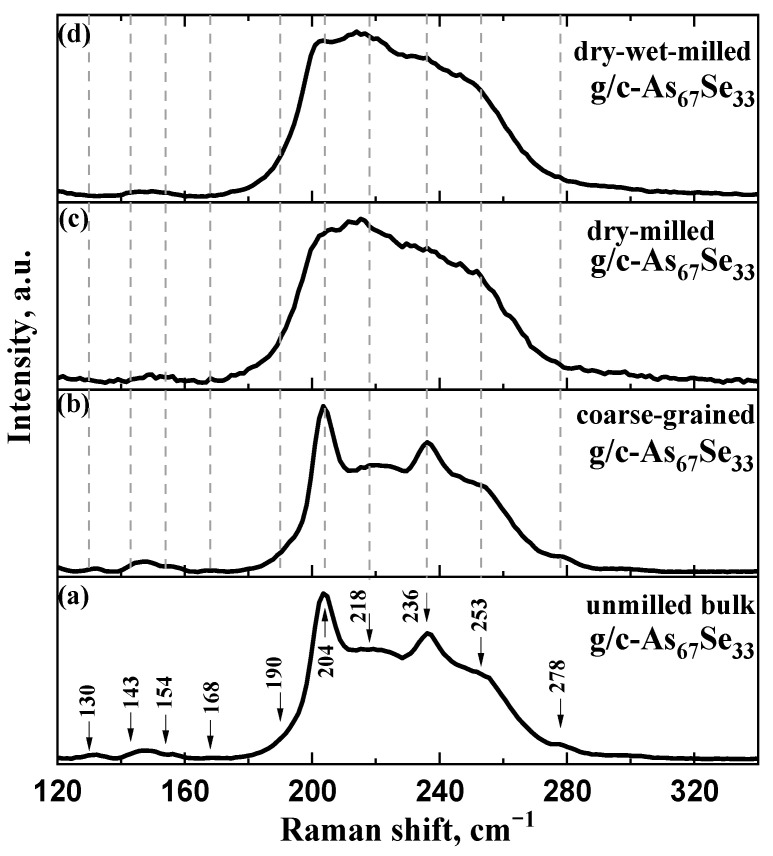
The normalized micro-RS spectra of MQ-derived g/c-As_67_Se_33_ reproduced in a sequence from the bottom to the top: (**a**) unmilled bulk pieces (unpelletized); (**b**) pelletized coarse-grained sample; (**c**) pelletized dry-milled sample; (**d**) pelletized dry–wet-milled sample. The most prominent features in the micro-RS spectrum of the bulk unmilled sample (**a**) are marked by vertical arrows, and traced by dotted lines to the respective micro-RS spectra of the pelletized samples (**b**–**d**).

To shed more light on the microstructure transformations in the examined arsenoselenide alloys, the micro-RS spectra were collected for these samples before and after the respective stages of their processing. For g/c-As_67_Se_33_ samples taken as unmilled bulk pieces and pelletized coarse-grained, dry-milled and dry–wet-milled samples, these micro-RS spectra normalized with respect to the amplitude of the dominant band are, respectively, reproduced in Figure 4a–d.

Firstly, we can confirm that palletization under the above conditions did not affect the micro-RS spectra of these samples. Indeed, this conclusion becomes obvious from a visual comparison of these spectra in Figure 4a,b collected for bulk pieces and pellets of MQ-derived g/c-As_67_Se_33_. Further, specifically for the Raman-active bands contributing to these spectra, a few low-frequency (at ~130, 143, 154, 168 and 190 cm^−1^) and high-frequency (at ~190, 204, 218, 236, 253, 278 cm^−1^) bands are resolved in the normalized micro-RS spectrum of unmilled g/c-As_67_Se_33_ (see Figure 4a). The high-frequency bands are ascribed to overlapped (mainly strong) bond-stretching modes of AsSe_3_ pyramids at 220–230 cm^−1^ [3,41,42] and thioarsenide molecules, such as As_4_Se_4_ (peaks at ~225 and 240 cm^−1^ supplemented by shoulder near 255 cm^−1^ [3,42]), As_4_Se_3_ (peaks at ~195, 205, 225, 240 and 255 cm^−1^ supplemented by shoulder near 280 cm^−1^ [42]) and As_4_ (strong peak at ~200–210 cm^−1^ [3,43,44]). The low-frequency bands (preferentially weak and medium) can be ascribed to the bond-bending modes of thioarsenide molecules, in part, As_4_Se_4_ (at ~145, 160 and 170 cm^−1^ [42,45,46]) and As_4_Se_3_ (at ~110, 135, 150, 160 and 170 cm^−1^ [42,45]). Thus, with respect to micro-RS spectroscopy, the appearance of the As phase is expected in MQ-derived g/c-As_67_Se_33_ samples due to the RS-active band at ~204 cm^−1^ ascribed to As_4_ thioarsenide molecules, while both As_4_Se_4_ and As_4_Se_3_ molecules are revealed via the strong band at ~236 cm^−1^ supplemented by a weak shoulder at ~278 cm^−1^ (see Figure 4a,b). The spectral region between these bands (220–230 cm^−1^) corresponds to the vibration modes of AsSe_3_ pyramids incorporated in a network via = As-Se-As = bridges. As a result, in g/c-As_67_Se_33_, the RS spectrum composed of overlapped high-frequency modes of thioarsenide molecules and network-forming fragments obeys a double-peak-shape characteristic with strong maxima at ~200 and ~240 cm^−1^ supplemented by a relatively suppressed hump at ~220–230 cm^−1^ and weak shoulder near ~280 cm^−1^ (for a comparison, see [3,27,42,45,46]).

These features revealed in micro-RS spectra of unmilled g/c-As_67_Se_33_ (shown in Figure 4a,b) are essentially broadened in this sample subjected to nanomilling in dry mode (see Figure 4c) or combined dry–wet mode (see Figure 4d). Such behavior speaks in favor of a nanomilling-driven molecular-to-network amorphization transition, which occurs due to the destruction of thioarsenide molecules followed by the incorporation of their remnants into the glass network undergoing a polyamorphic transition (amorphous-I-to-amorphous-II [47,48] or, alternatively, reamorphization transition [23]). These alloys, affected by defects generated under MM, become notably stressed in the nanomilled state, enhancing the calorimetric heat transfer responses, as compared with unmilled bulk samples [27].

**Figure 5:**
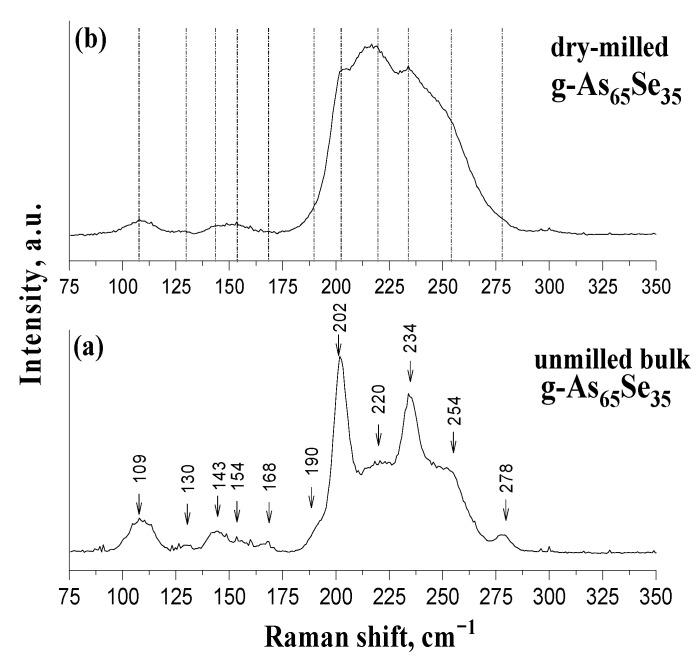
The normalized micro-RS spectra of MQ-derived g-As_65_Se_35_ in unmilled state (**a**) and after nanomilling in a single dry mode (**b**). The most prominent features in the micro-RS spectrum of the bulk sample (**a**) are marked by arrows and traced by dotted lines to that of dry-milled sample (**b**).

Noteworthily, the micro-RS spectroscopic identification of the rhombohedral (grey) As phase, which is expected due to two-fold degenerate E_g_ and A^1^_g_ modes near ~202 cm^−1^ and 254 cm^−1^ [49], is impossible in these alloys because of overlapping with the vibration bands of the arsenoselenide network. The same is true of nanocrystalline inclusions of the cubic arsenolite (As_2_O_3_) phase, which is expected in these alloys due to RS-active modes near ~190 cm^−1^ and ~270 cm^−1^ [44,49], which are also overlapped with As-Se network modes in this spectral range. Therefore, the absence of the above crystalline inclusions in the examined alloys does not change the principal appearance of their micro-RS spectra, as demonstrated in Figure 5 for purely glassy-like specimens (g-As_65_Se_35_) studied before and after dry MM.

Thus, atomic-specific microstructure transformations in the examined As-Se alloys driven by nanomilling are dominated by the polyamorphic (molecular-to-network) transitions of the existing MQ-derived amorphous phase (forming reamorphization channel), supplemented (especially under MM in a dry mode) by weak polymorphic (crystalline-to-amorphous) transitions of crystalline inclusions stabilized in these alloys under MQ (forming amorphization channel). Undoubtedly, because of the principal difference in the atomic packing of amorphous and crystalline phases in arsenoselenide alloys undergoing nanostructurization by MM, such transformations should be accompanied by the notable changes in atomic-deficient (free-volume) microstructure of these alloys.

### 2.2. Atomic-Deficient Microstructure of the Examined Arsenoselenides

**Figure 6:**
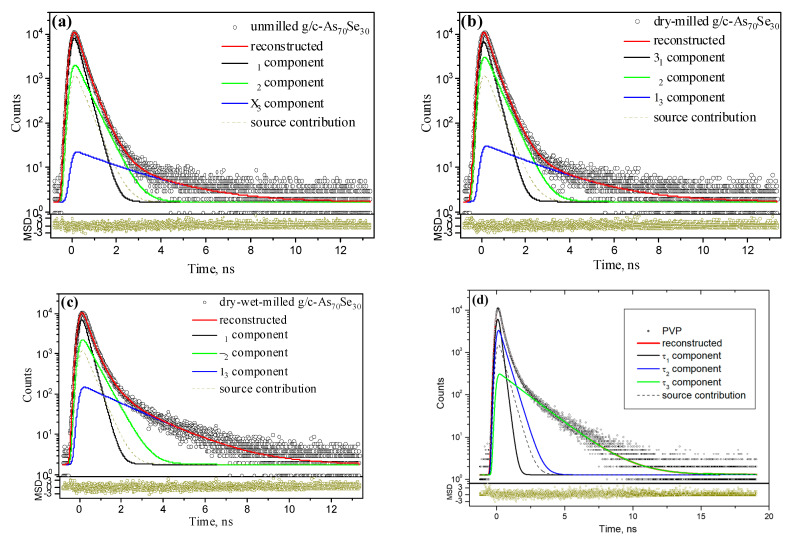
The raw PAL spectra of pelletized g/c-As_70_Se_30_ in unmilled state (**a**) and after nanomilling in dry mode (**b**) and combined dry–wet mode (**c**) as compared with the spectrum of PVP pelletized under the same conditions (**d**). The collected PAL spectra are reconstructed from unconstrained three-term fitting and reproduced at background of source contribution with bottom insets showing statistical scatter of variance. The occupation of “tail” states in unmilled and dry-milled samples grows notably under transition to dry–wet-milled sample approaching that in the pelletized PVP.

The raw PAL spectra reconstructed from unconstrained three-term fitting for g/c-As_70_Se_30_ alloys in the unmilled (bulky) state and after the respective stages of nanomilling are depicted in Figure 6, and they are compared with the spectrum of ‘pure’ PVP pelletized under the same conditions [50]. The applicability of this fitting procedure is proved by a scatter of variance (representing minimal statistically weighted least-square deviation between experimental and theoretical curve built of three exponentials) tightly grouped along the time axis in the bottom insets of Figure 6. Similar PAL spectra (not reproduced in Figure 6) were collected for other pelletized arsenoselenides. The best-fit PAL spectra parameters (component lifetimes *τ_i_* and intensities *I_i_*, *I* = 1, 2, 3) for arsenoselenide samples and PVP (pelletized under the same conditions [50]) are gathered in Table 2, and positron-trapping and Ps-decaying modes derived from these PAL spectra within two-state simple-trapping model (STM) [28,29] ignoring the Ps-decaying contribution are listed in Table 3.

Just from a visual inspection of Figure 6, it is clear that occupation of the ‘tail’ states related to bound positron–electron (positronium, Ps) states grows notably in bulk and dry-milled As-Se alloys under the transition to dry–wet-milled samples, approaching that in ‘pure’ PVP. This means that the transition from preferential annihilation via positron trapping to Ps decays. The PAL spectra of both unmilled and dry-milled arsenoselenides reveal a defect-specific lifetime of *τ_2_*~0.36–0.39 ns (Table 2), the value characteristic for multiatomic (bi-, tri-, quadruple) vacancies in amorphous As-Se [29,51,52,53,54,55]. The third component in the collected PAL spectra of these samples with *τ_3_*~2.1 ns and small fractional free volume below *f_v_*~0.3 can be ascribed to holes with a radius of *R_3_*~0.30 nm (Table 3). Because of the small input from the Ps-decay channel, the derived defect-free bulk lifetime *τ_b_* approaches ~0.27 ns for the unmilled g-As_65_Se_35_ sample and somewhat lower, ~0.25 ns, for the g/c-As_70_Se_30_ sample. Noteworthily, the *τ_b_* lifetime in crystalline arsenic triselenide is ~0.24 ns [51], which increased to ~0.28–0.29 ns in the amorphous matrix [29,52,53,54,55]. Therefore, positron trapping in unmilled and dry-milled arsenoselenides is expected to occur preferentially in the disordered As-Se network.

With the transition to samples pelletized from dry–wet-milled arsenoselenides (representing, in fact, PVP-capped nanocomposites [24,25,26]), the location of the preferential annihilation channel is changed. As follows from Table 2, the *τ_2_* lifetime is notably increased in dry–wet-milled samples approaching ~0.43 ns (the value characteristic for positron trapping in large vacancies in As-Se matrix [51,52]), while the *τ_3_* lifetime is decreased to ~1.9 ns (closer to Ps-related lifetime in PVP, *τ_3_* = 1.867 ns [50]), resulting in an evidently increased average lifetime, *τ_av._*~0.38 ns. It means that additional free volume contributes to positron trapping sites in wet-milled samples while disappearing in Ps-hosting holes. Nevertheless, the more than three-times increased third component intensity (*I_3_*) speaks in favor of the fragmentation of Ps-decay sites, leading to fractional free volume *f_v_*~0.8 (Table 3). These changes are counterbalanced by decreased *I_2_*, preferring (under criterion [56]) an increased Ps-formation probability in the transition from dry-milled to dry–wet-milled arsenoselenide samples.

**Figure 7:**
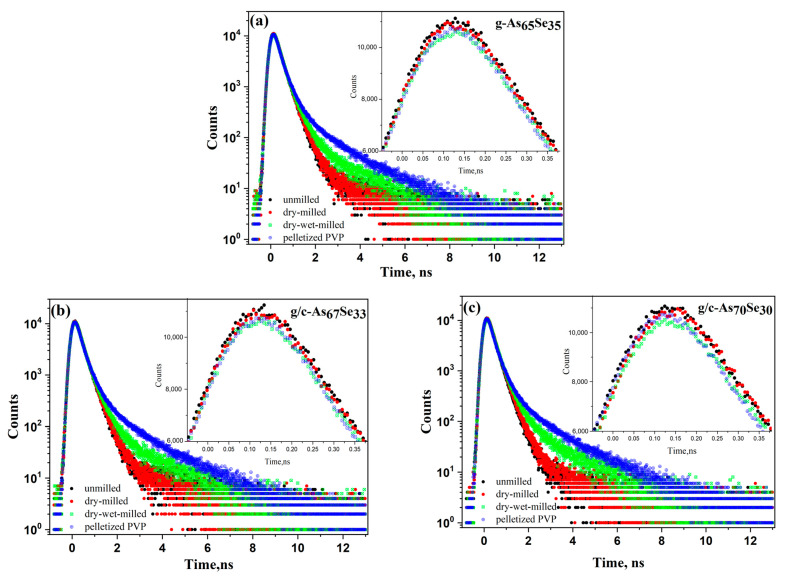
The overlapping of the PAL spectra in the examined arsenoselenides g-As_65_Se_35_ (**a**), g/c-As_67_Se_33_ (**b**) and g/c-As_70_Se_30_ (**c**) pelletized before nanomilling (black points) and after nanomilling in dry mode (red points) and dry–wet mode (green points) as compared with the PAL spectrum in the PVP sample pelletized under the same conditions (blue points). The insets show a nearly invariant tendency in the PAL spectra peaks depressed in the right wing after nanomilling in dry–wet mode due to moderated Ps-formation probability and slightly changed average positron lifetime *τ_av_*. The changes in the PAL spectra tails of unmilled, dry- and dry–wet-milled samples are due to increase in density of o-Ps hosting holes. There is no evident empty gap between the PAL spectra tails for dry-milled and dry–wet-milled glassy samples as compared with glassy-crystalline samples caused by changes in Ps decaying states under transition to annihilation in PVP-bearing medium.

Therefore, the Ps decay in the PVP-capped nanocomposites occurs rather in ‘pure’ PVP environment than in the amorphous As-Se matrix. A comparative presentation of the respective PAL spectra for these arsenoselenide alloys and PVP shows a spectacular trend in their superposition, specifically the compositionally tuned behavior in the overlapped PAL spectra highlighted in Figure 7. Smooth monotonic changes are observed in both PAL spectra peaks and tails in these samples undergoing nanostructurization in the transition between states characteristic for unmilled (or dry-milled) and wet-milled samples. Similar behavior was also observed in other As-Se alloys from the glass-forming region [24,25,26]. In contrast, in the examined glassy and glassy-crystalline alloys from the border of the glass-forming region, an almost invariant tendency is observed in the PAL spectra peaks depressed on the right wing after wet milling due to the moderated Ps formation probability in PVP water solution and slightly changed average positron lifetime (*τ_av_*). These changes in the PAL spectra tails for unmilled, dry-milled and dry–wet-milled samples are due to the increased density of Ps-hosting holes, defined merely by the *I_3_* intensity. There is no evident empty gap between these spectra tails for dry-milled and dry–wet-milled g-As_65_Se_35_ samples (Figure 7a), as compared with glassy-crystalline g/c-As_67_Se_33_ or g/c-As_70_Se_30_ samples (see Figure 7b,c) caused by changes in the Ps-decaying channel under the transition from amorphous As-Se to the PVP-bearing environment. The richer variety of positron traps in glassy-crystalline alloys with *τ_2_*~0.43 ns (see Table 2) facilitates the formation of such an empty gap, as follows from the comparison between respective PAL spectra in Figure 7.

To shed more light on the examined As-Se alloys undergoing nanomilling-driven volumetric changes, the PAL spectra of dry-milled samples are considered with respect to these spectra in wet-milled samples employing revised positron lifetime analysis within the Positronics approach [28,29]. The trapping conversion parameters derived from such comparisons are presented in Table 4. Positive values of the first and second component intensities (*I*_n_ and *I_int_*) in the reconstructed PAL spectrum of the heterogeneous medium undergoing volumetric nanostructurization, along with the well-defined component inputs, testify that Ps-hosting holes in these nanocomposites (g-As_65_Se_35_/PVP, g/c-As_67_Se_33_/PVP and g/c-As_70_Se_30_/PVP) are transformed in positron traps typical for dry-milled samples. The defect-specific lifetimes *τ_int_*~0.36–0.38 ns (Table 4) show that positron traps are large interfacial free-volume voids close to the multiatomic vacancies in the amorphous As-Se matrix [51,52,53,54,55]. This finding agrees with (*τ_int_*-*τ_b_^NP^*) ~0.09–0.11 ns and *τ_int_*/*τ_b_^NP^*~1.4, indicative of large vacancies in chalcogenide compounds [57]. The overall conversion process, i.e., appearance of positron traps with *τ_int_*~0.36–0.38 ns instead of Ps-hosting holes with *τ_3_*~2.1 ns, occurs in an environment with an effective bulk lifetime of *τ_b_^NP^*~0.29 ns, which is above *τ_b_* in crystalline arsenic selenide (~0.240 ns [51]) approaching the value characteristic for glassy As-Se (~0.28–0.29 ns) [52,53,54,55]. Therefore, we can reasonably speculate that a heterogeneous medium accommodates the products of direct Ps-to-positron conversion, i.e., unoccupied free-volume spaces where positron traps (typical for dry-milled As-Se alloys) appear instead of Ps-decaying holes (typical for PVP-stabilized nanocomposites) is the amorphous As-Se matrix, slightly enriched in some impurity products such as arsenic oxide.

### 2.3. Molecular–Network Disproportionality at the Border of the Glass-Forming Region in As-Se System via Quantum–Chemical Cluster Modelling

In substances like thioarsenide As_4_Se_n_-bearing compounds in the As_x_Se_100−x_ system, effects from the selective localization of positron-trapping and Ps-hosting sites are strongly enhanced due to the glassy-crystalline environment around intrinsic free-volumes elements (such as voids, atomic vacancies, vacancy-like clusters, pores, cracks, etc.) acting as potential setting places for trapped positrons and bound positron–electron (positronium, Ps) states [28,29]. Following the famous prediction of Blachnik and Wickel in 1984 on the thermal decomposition of A_4_B_3_ cage-like molecules into A_4_B_4_ ones and unidentified amorphous products [58], it has been proved recently [59] that nanomilling facilitates the decomposition of As_4_Se_3_ molecules in arsenoselenides within the glass-forming region in the vicinity of *x* = 57 (dimorphite-type composition), resulting in a reamorphized glass structure enriched in realgar-type As_4_Se_4_ molecules (prevailing near *x* = 50) and network remainders close to tetra-arsenic biselenide As_4_Se_2_ (dominated at *x* = 67). Since there are no molecular thioarsenides among products of devitrification in arsenoselenides from the border of the glass-forming region in the As_x_Se_100−x_ system (65 < *x <* 70), it seems reasonable to assume that crystallization processes are a result of the spontaneous (under MQ) or activated (under thermal or mechanical energy) decomposition of some network derivatives near As_4_Se_2_ composition (*x* = 67).

Cluster modelling of the molecular and network conformations was performed previously with the help of the ab initio quantum–chemical modelling algorithm CINCA [60,61] for As_x_Se_100−x_ alloys with crystalline counterparts, such as As_2_Se_3_ (*x* = 40) [62,63,64], As_4_Se_4_ (*x* = 50) [23] and As_4_Se_3_ (*x* = 57) [59]. On this basis, let us examine the forming energies (*E_f_*) among As_4_Se_n_ thioarsenide clusters (*n* = 6–0), covering the glass-forming border in the As_x_Se_100−x_ system (65 < *x* < 70), positioned around tetra-arsenic biselenide As_4_Se_2_ (*x* = 67, *n* = 2) and beyond this composition approaching tetra-arsenic As_4_ (*x* = 100, *n* = 0) and tetra-arsenic hexaselenide As_4_Se_6_ (*x* = 40, *n* = 6), the latter being equivalent to As_2_Se_3_.

In a group of tetra-arsenic hexaselenide (As_4_Se_6_) clusters [62,63,64], a strong network-forming trend clearly dominates, resulting in layered arsenoselenide 2D structures based on corner-shared AsSe_3/2_ pyramids with average cluster-forming energy, *E_f_* ~ −72.619 kcal/mol [63], which is even better than the *E_f_* energy of the single trigonal AsSe_3/2_ pyramid (−72.309 kcal/mol) [61]. The prevailing canonical chain-crossing model [1,2,3] in As_x_Se_100−x_ glasses at *x* < 40 is governed by decomposition on two-cation Se-rich clusters (under-constrained or floppy in view of number of constraints n_c_ calculated after the Phillips–Thorpe algorithm [65,66,67] less than space dimensionality, 3.0) and As_2_Se_3_ clusters (optimally constrained, rigid but not stress, n_c_ = 3.0) [61]. Disproportionality analysis shows that As_2_Se_3_-bearing network structures are subjected to intrinsic decomposition on Se-rich chain-like clusters and As-rich As_2_Se_4/2_ clusters based on homonuclear (As-As) bonding between the AsSe_3/2_ pyramids, acting as pre-cursors of As_4_Se_4_ thioarsenide molecular entities [61,62,63].

Thus, in a group of tetra-arsenic tetraselenide thioarsenides, the strong molecular-forming tendency becomes dominated with under-constrained (*n_c_* = 2.875) realgar-type As_4_Se_4_ cage molecules of *D*_2d_ symmetry, with *E_f_* ~ −72.713 kcal/mol, which is 0.40 kcal/mol better than *E_f_* in the AsSe_3/2_ unit [23]. Among network derivatives from this As_4_Se_4_ molecule, the most plausible is an optimally constrained cluster (*n_c_* = 3.0) that appeared due to a single break in one of four Se atom positions (*E_f_* ~ 0.25 kcal/mol), which can be labelled as x1-As_4_Se_4_ following the accepted CINCA nomenclature [60,61].

Among tetra-arsenic triselenide thioarsenides (corresponding to *x* = 57), the dimorphite-type As_4_Se_3_ cage molecule (under-constrained in view of *n_c_* = 2.71) composed of a triangular-pyramidal (As_3_)-As conformation is most plausible, possessing *E_f_* ~ 0.33 kcal/mol (with respect to AsSe_3/2_ pyramid) [59]. All seven atoms in this molecule of *C_3v_* symmetry (isostructural with the molecule refined in α- and β-modifications of mineral dimorphite As_4_S_3_ by Whitfield [68,69]) are located in the same sphere, resulting in a specific 0D structure with low heat transfer and strong thermal expansion responses, which are accepted as characteristic features of the plastically crystalline As_4_Se_3_ modification [58,70]. Other molecular clusters of this type and their network-forming derivatives reconstructed by the break in Se atom positions are unfavorable, as compared with this dimorphite-type As_4_Se_3_ molecule [59], and, therefore, cannot be taken into consideration under comprehensive analysis of molecular–network disproportionality in arsenoselenides of similar chemical compositions.

**Figure 8:**
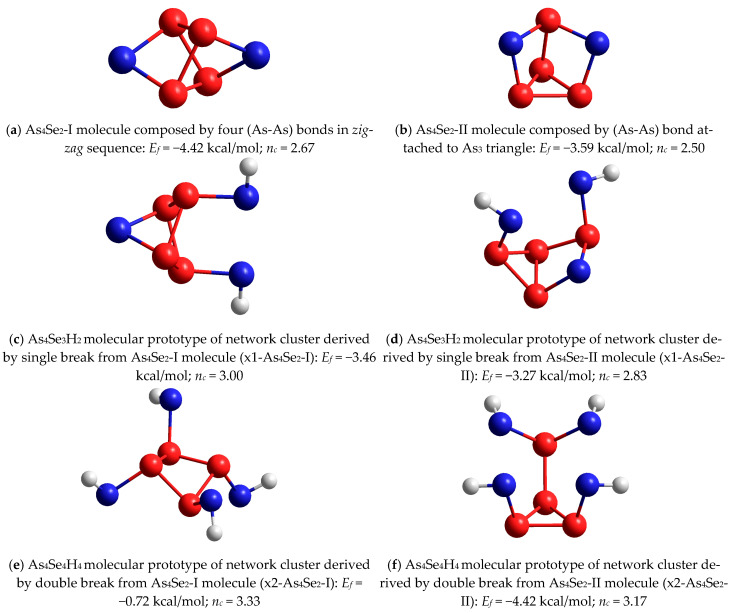
The ball-and-stick presentation of optimized configuration of tetra-arsenic biselenide thioarsenide As_4_Se_2_-I molecule composed by four (As-As) bonds in *zig-zag* sequence (**a**) and As_4_Se_2_-II molecule composed by (As-As) bond attached to As_3_ triangle (**b**), as compared with As_4_Se_3_H_2_ and As_4_Se_4_H_4_ molecular prototypes of network clusters derived from these molecules by single (x1-As_4_Se_2_-I—(**c**), x1-As_4_Se_2_-II—(**d**)) and double (x2-As_4_Se_2_-I—(**e**), x2-As_4_Se_2_-II—(**f**)) breaking in available Se atom positions. The cluster-forming energies *E_f_* are given in respect to AsSe_3/2_ pyramid (*E_f_* = −72.309 kcal/mol [60]). The H, Se and As atoms are, respectively, grey-, blue- and red-colored, and chemical bonds between atoms are denoted by respectively colored sticks. The average number of constraints *n_c_* is given following the Phillips-Thorpe constraint-counting algorithm [65,66,67].

In a group of tetra-arsenic biselenide (As_4_Se_2_) thioarsenides (*x* = 67), both molecular entities, the As_4_Se_2_-I cluster with *n_c_* = 2.67 composed of four (As-As) bonds in a *zig-zag* sequence (see Figure 8a) and the As_4_Se_2_-II cluster with *n_c_* = 2.50 composed of a (As-As) bond attached to the As_3_ triangle (Figure 8b), are unfavorable in view of *E_f_* energy, approaching −4.42 kcal/mol and −3.59 kcal/mol, respectively. The same concerns network-forming clusters derived from these molecules by breaking in one of the Se atom positions, with both As_4_Se_3_H_2_ molecular prototypes reproduced in Figure 8c,d. But this situation cardinally changed in the transition to network clusters derived from these molecules by double breaking in both Se atom positions, with the H-saturated molecular prototypes of these clusters shown in Figure 8e,f. Surprisingly, the network-forming cluster (x2-As_4_Se_2_-I) derived from the As_4_Se_2_-I molecule, maintaining the closed tetragon-like As_4_ arrangement built of four (As-As) bonds (Figure 8e), has *E_f_* ~ −0.72 kcal/mol, which is the best among all clusters in this compositional range [59]. Because of one small ring involved, the glassy network built of such clusters is rigid and stressed (*n_c_* = 3.33). These clusters facilitate the decomposition of dimorphite-type As_4_Se_3_ molecules into realgar-type As_4_Se_4_ ones, accompanied by the extraction of some amorphous products close to As_4_Se_2_ [59].

**Figure 9:**
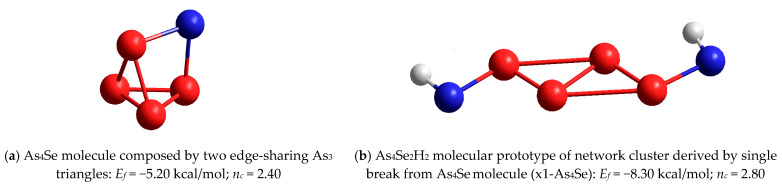
The ball-and-stick presentation of optimized configuration of tetra-arsenic monoselenide As_4_Se molecule composed by two edge-sharing As_3_ triangles (**a**), as compared with As_4_Se_2_H_2_ molecular prototype of network-forming cluster derived from this molecule by breaking in Se atom position x1-As_4_Se (**b**). The terminated H atoms are grey-colored, Se and As atoms are respectively blue- and red-colored, chemical bonds between atoms are denoted by, respectively, colored sticks.

In a group of tetra-arsenic monoselenide (As_4_Se) thioarsenides (*x* = 80), where only two clusters are possible, the resolution is very simple. Any of these clusters, neither molecular As_4_Se composed of two edge-sharing As_3_ triangles (Figure 9a) nor network-forming x1-As_4_Se reproduced from this molecule by breaking in the Se atom position (see Figure 9b), can be stabilized in a realistic structure because of the very unfavorable cluster-forming energies *E_f_* far beyond −5 kcal/mol.

**Figure 10:**
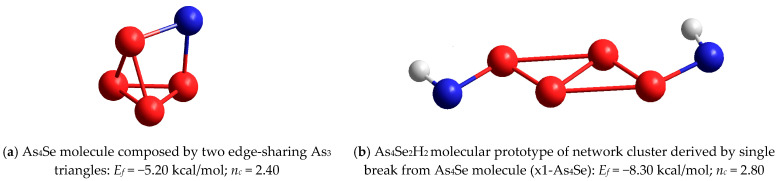
The ball-and-stick presentation of optimized configuration of regular pyramid-shaped tetra-arsenic As_4_ thioarsenide molecule (**a**) and As_6_H_6_ molecular prototype of network-forming cluster derived by distortion from this molecule in a form of flattened pyramidal-shaped unit, which composes two-dimensional double-layer network of chair-configurated six-fold rings of As atoms (As_6⋅(2/3)_ = As_4_). The H and As atoms are respectively grey- and red-colored, and chemical bonds between atoms are denoted by respectively colored sticks.

Thus, in a group of tetra-arsenic As_4_ clusters (corresponding to *x* = 100 in As_x_Se_100−x_ system), the situation is non-trivial and complicated. The most stable amorphous and crystalline structures related to As polymorphs can be reconstructed from two principal building blocks typical for gaseous and condensed As states, these being, respectively, (i) the thioarsenide molecular cluster in the form of a regular four-atom pyramid-shaped As_4_ tetrahedron and (ii) the thioarsenide-type network-forming cluster in the form of a flattened pyramid-shaped unit derived through distortion from this regular As_4_-tetrahedral-like molecule by breaking in one of three As-As bonds at each As atom composed of a two-dimensional double-layer network of chair-configurated six-fold rings (As_6⋅(2/3)_ = As_4_). These blocks within the arrangement of the three nearest neighbors can be differentiated by the *E_f_* energies derived from the CINCA modelling, while distortion pathways beyond the three nearest neighbors explain the emergence of As allotropes in orthorhombic and rhombohedral structures [30], but this specificity cannot be accounted for within this cluster modelling routine. The under-constrained configuration of the tetrahedral As_4_ molecule (*n_c_* = 2.25) in Figure 10a is found to be very unfavorable in realistic structures in view of *E_f_* ~ −4.31 kcal/mol. This finding is in line with the fact that yellow As (also named insulator, γ-As) consisting of As_4_ tetrahedra exists only in the gaseous state [71,72]. In contrast, the *E_f_* energy of over-constrained network clusters (*n_c_* = 4.5) composed of a double-layer honeycomb structure of chair-configurated (As_6⋅(2/3)_ = As_4_) rings (see Figure 10b) typical of both rhombohedral (grey or metallic, α-As) and orthorhombic (black or semiconducting, β-As) allotropes [30,31,71,72] seems very promising in view of *E_f_* ~ −2.46 kcal/mol. This finding explains the appearance of the rhombohedral As phase as the most stable allotrope under crystallization in MQ-derived As-Se alloys.

**Figure 11:**
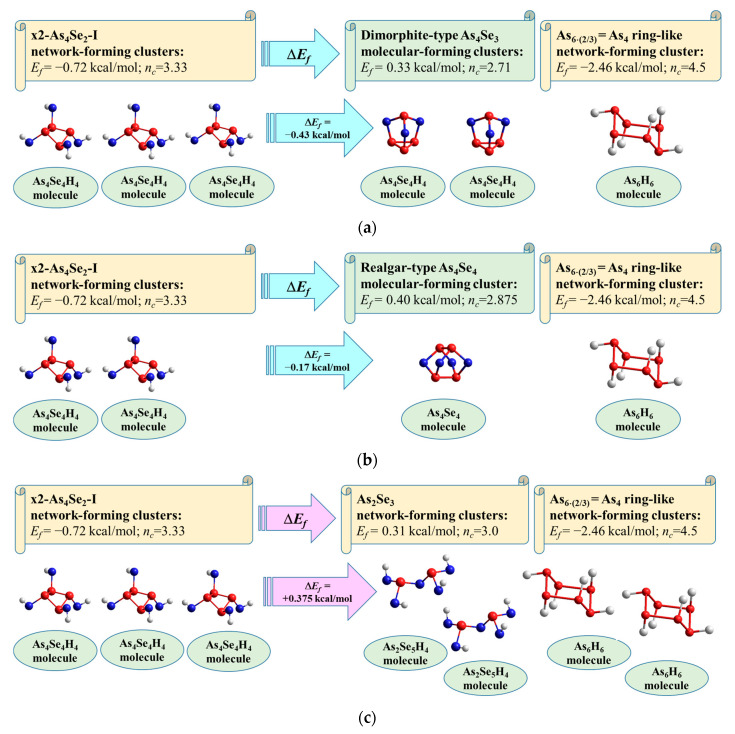
Three decomposition scenarios of x2-As_4_Se_2_-I network-forming clusters governing molecular-network disproportionality in tetra-arsenic biselenide As_4_Se_2_-bearing arsenoselenides: (**a**)—spontaneous decomposition under Δ*E_f_* = –0.43 kcal/mol; (**b**)—spontaneous decomposition under Δ*E_f_* = –0.17 kcal/mol; (**c**)—activated decomposition under Δ*E_f_* = +0.375 kcal/mol. The terminated H atoms are grey-colored, Se and As atoms are, respectively, blue- and red-colored, and chemical bonds between atoms are denoted by the respective colored sticks.

Let us parameterize molecular–network disproportionality at the border of the glass-forming region in the As-Se system (close to As_4_Se_2_) based on the cluster-forming energies *E_f_* calculated for the components of most expected decomposition scenarios, sketched in Figure 11.

The first scenario (Figure 11a) involves the decomposition of x2-As_4_Se_2_-I network-forming clusters (shown in Figure 8e) on dimorphite-type As_4_Se_3_ molecules [59] supplemented by the extraction of As_6⋅(2/3)_ ring-like network-forming clusters (see Figure 10b), which occurs with a negative energetic barrier (Δ*E_f_*) corresponding to the spontaneous decomposition of the examined arsenoselenide alloy under MQ:

3⋅(x2-As_4_Se_2_-I) → 2 As_4_Se_3_ + As_6⋅(2/3)_ + (Δ*E_f_* = −0.43 kcal/mol) (1)

By removing down from As_4_Se_2_ stoichiometry, the second disproportionality scenario involving the spontaneous decomposition of x2-As_4_Se_2_-I network-forming clusters (Figure 8e) on realgar-type As_4_Se_4_ molecules [23] supplemented by the extraction of As_6⋅(2/3)_ ring-like network clusters (Figure 10b) occurring also with a negative barrier becomes clear (see Figure 11b):

2⋅(x2-As_4_Se_2_-I) → As_4_Se_4_ + As_6⋅(2/3)_ + (Δ*E_f_* = −0.17 kcal/mol) (2)

Further approaching stoichiometric arsenic triselenide As_2_Se_3_, the third molecular–network disproportionality scenario, which involves the decomposition of x2-As_4_Se_2_-I network-forming clusters (Figure 8e) on layer-type As_2_Se_3_ network clusters composed of two corner-shared AsSe_3/2_ pyramids (as shown in [61,62]) supplemented by the extraction of As_6⋅(2/3)_ ring-like network clusters (Figure 10b), occurring with a positive energetic barrier, can be validated (see Figure 11c):

3⋅(x2-As_4_Se_2_-I) → 2⋅As_2_Se_3_ + 2⋅As_6⋅(2/3)_ + (Δ*E_f_* = +0.375 kcal/mol) (3)

The positive value of the energetic barrier Δ*E_f_* in Equation (3) means that this decomposition scenario cannot be realized spontaneously but, rather, as activated thermal or milling-driven decomposition.

The spontaneous nature of molecular–network disproportionality in As_x_Se_100−x_ alloys from the border of the glass-forming region (65 < *x* < 70), obeying reactions (1) and (2), is proved by a large amount of molecular thioarsenides in these alloys stabilized under MQ with respect to the micro-RS spectra of unmilled samples (see Figure 4a,b and Figure 5a) and the crystalline inclusions of the rhombohedral As phase in these samples revealed by the XRPD analysis (see Figure 1, Figure 2 and Figure 3). Thus, from this disproportionality analysis, the compositional location of the border of the glass-forming region in the As-Se system nearby the As_4_Se_2_ thioarsenide composition (CN = 2.67) becomes completely understandable.

The nanomilling-driven molecular-to-network transition in As_4_Se_2_-bearing alloys is described by decomposition reaction (3), with a positive energetic barrier (Δ*E_f_* = 0.375 kcal/mol) comparable to the decomposition of As_4_Se_3_ molecules in As_4_Se_4_ ones and accompanying amorphous products (Δ*E_f_* = 0.41 kcal/mol) [59]. The transition towards more network conformations is accompanied by interplay between medium-range structure levels in As-Se alloys subjected to nanomilling (see Figure 1) and the respective broadening of their micro-RS spectra (see Figure 4c,d and Figure 5b). Since the remnants of thioarsenides destructed under grinding interact with oxygen, especially in PVP water solution, the cubic arsenolite As_2_O_3_ phase is intensively formed in these alloys subjected to nanomilling in combined dry–wet mode (see Figure 3). Because of the enhanced heterogeneity in the atomic-deficient microstructure, the superposition of the PAL spectra collected for PVP and MQ-derived umilled, dry-milled and dry–wet-milled samples shows a spectacular smoothly decaying trend over superimposed PAL spectra peaks and tails (highlighted in Figure 7).

## 3. Materials and Methods

### 3.1. Preparation, Preliminary Testing and Nanomilling of the Examined Arsenoselenides

The border of the glass-forming region in As_x_Se_100−x_ system from As-rich side is exemplified by MQ-derived alloys compositionally deviated around tetra-arsenic biselenide (As_4_Se_2_) thioarsenide stoichiometry, corresponding in coordination number CN = 2.67, these being under-stoichiometric glassy g-As_65_Se_35_ (CN = 2.65), stoichiometric glassy-crystalline g/c-As_67_Se_33_ (or g/c-As_4_Se_2_) and over-stochiometric glassy-crystalline g/c-As_70_Se_30_ (CN = 2.70). These alloys were synthesized via vibrational MQ from elemental precursors (As and Se of 5N purity), as described in more detail elsewhere [11,12,15,18]. The density *ρ* (±0.005 g⋅cm^−3^) of coarse-grained pieces of samples defined by the Archimedes displacement method in ethanol was lower in the glassy state (4.448 g⋅cm^−3^ for g-As_65_Se_35_) and higher in the glassy-crystalline state, approaching 4.654 g⋅cm^−3^ for g/c-As_70_Se_30_. The mean inter-atomic spacing *d_s_^m^* derived from the density of g/c-As_67_Se_33_ alloy was ~3.79 Å.

Mechanical treatment of the prepared alloys was performed in two stages.

Firstly, the MQ-derived ingots were subjected to nanostructurization by dry MM in a protective argon atmosphere in a planetary ball mill Pulverissete 6 (Fritsch, Idar-Oberstein, Germany). This grinding route was performed under a rotational speed of 500 rpm in 250 mL tungsten carbide chamber loaded with 50 tungsten carbide balls (each 10 mm in diameter) using 3 g of preliminary coarse-grained material sieved under 200 μm. Then, the fine-grained powder was pelletized in a stainless-steel die via ~0.7 GPa compressing, stabilizing dry-milled samples for further research.

In the second stage, part of the powder (prepared in a dry mode) was subjected to additional attrition in wet mode using a laboratory MiniCer mill (Netzsch, Germany), operational under a rotational speed of 3500 rpm. This 90-minute route was performed in 300 mL of 0.5% PVP water solution, milling shaft being loaded on 85% with yttrium-stabilized ZrO_2_ balls (each 0.6 mm in diameter). The PVP of ~40,000 g⋅mol^−1^ molecular weight purchased from Sigma-Aldrich Co. LLC (St. Louis, MO, USA) was used to prepare compositionally authentic nanosuspensions. Finally, they were dried at 70 °C and pelletized via compressing (under ~0.7 GPa) in stainless-steel die, stabilizing a set of the As_x_Se_100−x_/PVP nanocomposites as plane-parallel disc-like pellets (~6 mm in diameter, ~1 mm in thickness), the most suitable shape for micro-RS and PAL measurements.

**Figure 12:**
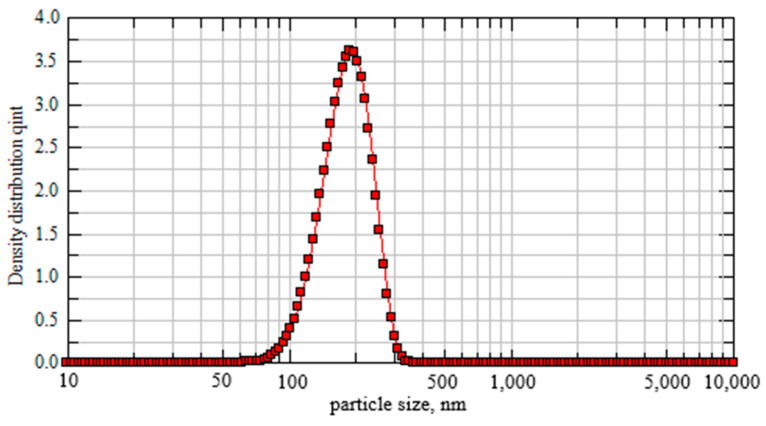
The unimodal particle size distribution in nanosuspension of MQ-derived g-As_65_Se_35_ showing the parameters (x50) and (x99), respectively, approaching ~182 nm and ~291 nm.

The particle size distribution was recognized for nanosuspensions of powdered As-Se alloys, employing photon cross-correlation spectroscopy with Nanophox particle size analyzer (Sympatec, Clausthal-Zellerfeld, Germany). The unimodal particle size distribution centered around ~180–190 nm was monitored in all cases. As an example, the particle size distribution in the suspension of nanomilled g-As_65_Se_35_ showing (x50)~182 nm (meaning that 50% of particles are smaller than 182 nm) and (x99)~291 nm (meaning that 99% of particles are smaller than 291 nm) is shown in Figure 12.

### 3.2. Atomic-Specific Phase Composition and Medium-Range Structure via Xrpd Analysis

The phase composition and medium-range structure of the arsenoselenides were recognized with the XRPD analysis using the STOE STADI P diffractometer operational in transmission mode of Cu Kα1-radiation (for details of the XRPD analysis, see [20,21,22,23,24]).

The phase diagram of As-Se alloys contains three stable crystalline compounds corresponding to different thioarsenide As_4_Se_n_ stoichiometry, such as arsenic triselenide As_2_Se_3_ equivalent to tetra-arsenic hexaselenide As_4_Se_6_ (*n* = 6), arsenic monoselenide AsSe (tetra-arsenic tetraselenide As_4_Se_4_, *n* = 4) and tetra-arsenic triselenide As_4_Se_3_ (*n* = 3), existing as two normal crystalline polymorphs identified by Bastow and Whitfield [32] and nominated by Blachnik and Wickel [58] as ambient-temperature monoclinic α-As_4_Se_3_ and high-temperature orthorhombic α′-As_4_Se_3_. Under heating above 412 K, the α-As_4_Se_3_ transforms in α′-As_4_Se_3_ phase, and under further heating above 447 K, the latter transforms into plastically crystalline β-As_4_Se_3_ and unidentified amorphous phase, while only normal crystalline α′-As_4_Se_3_ phase can be obtained at ambient temperature by MQ [58]. Therefore, processing of the XRPD patterns was performed to recognize phase composition of the arsenoselenides using the data for As-Se polymorphs available from the known databases [73,74] and other resources, in part, the JCPDS card No. 65–2365 for monoclinic As_2_Se_3_ (space group s.g. P2_1_/n, structure type s.t. α-As_2_S_3_, orpiment [35,36]), No. 73–0465 for trigonal Se (s.g. P3121 [37]), No. 71–0388 for monoclinic As_4_Se_4_ (s.g. P2_1_/n, s.t. α-As_4_S_4_, realgar [33,34]), No. 04–4979 for orthorhombic As_4_Se_3_ (s.g. Pnma, s.t. α-As_4_S_3_, dimorphite [32]), No. 72–1048 for rhombohedral (grey) As or α-As (s.g. *R*$\bar{3}$*m* [30,31]), No. 36–1490 for cubic arsenolite As_2_O_3_ (s.g. *Fd3m* [38]).

The amorphous phase in the examined alloys was identified due to diffuse peak halos in their XRPD patterning, in part, the FSDP (the first sharp diffraction peak), which is believed to be a signature of structural entities forming intermediate-range ordering in glass over a few tens Å reproduced in a reciprocal space near scattering vector *Q*_1_ ~(1–1.5) Ǻ^−1^ [75], and the SSDP (the second sharp diffraction peak, in terms of Elliott [76]) or PDP (the principal diffraction peak, in terms of Zeidler and Salmon [77]) serving as signature of extended-range order at *Q*_2_ ~(1.8–2.2)Ǻ^−1^. In the XRPD patterning of As-rich As_x_Se_100−x_ alloys (*x* > 40), the FSDP-related peak halo at ~(15–22)°2*θ* reflects correlations between some polyhedrons like thioarsenide As_4_Se_n_ molecules, while the SSDP-related halo shifted to ~(28–33)°2*θ* reflects orientational specificity of these polyhedrons ascribed to the second-order pair atomic correlations close to the mean inter-atomic spacing, *d*_s_^m^ [35]. At ~(50–60)°2*θ* (equivalent to *Q*_3_ ~3.3–4.0 Ǻ^−1^), the third diffraction peak (TDP) is observed as manifestation of shortest (~2.1–2.3 Ǻ) interatomic separation in a glass [35,77]. Thus, the XRPD measurement reveals three-peak structure of the patterns, which reflects a succession of single pairwise correlations defined by *Q*_3_ = *Q*_TDP_ and multi-pairwise correlations determined by *Q*_1_ = *Q*_FSDP_ and *Q*_2_ = *Q*_SSDP_ responsible for medium-range ordering [77].

Arrangement of diffuse peak halos in the XRPD patterns responsible for amorphous phase was analyzed using the STOE WinXPOW 3.03 [78] and PowderCell 2.4 [79] program packages, following normalization procedure with respect to the maximum. The error bar in the peak halo position (2*θ*) and full width at half maximum (FWHM) was not worse, ±0.05°2*θ*; the scattering vector position and width were calculated as *Q* = (4π/*λ*)⋅sin*θ* and Δ*Q* = (4π/*λ*)⋅sin(FWHM/2), respectively. The characteristic distance *R* (the spacing of peak halo responsible quasi-periodicity) and the correlation length *L* over which this quasi-periodicity were maintained and defined as for the Bragg diffraction (*R* = 2π/*Q*, *L* = 2π/Δ*Q*). Within modified microcrystalline approach [20,21,22,23,24], the diffuse peak-halo arrangement in the XRPD patterning in chalcogenide glass was also treated as arising from diffraction of coordination spheres, i.e., the shortest inter-atomic distances like in randomly packed multiparticulate systems (see [80] and literature therein), when the XRPD is governed by the Ehrenfest relation [81]:

2*d_s_*⋅sin *θ* = 1.23⋅λ (4)

where *d_s_* is the average distance between scatterers (the radius of the coordination sphere). Noteworthily, the error bar in the above parameters (*R*, *L*, *d_s_*) does not exceed ±0.1 Å.

### 3.3. Atomic-Specific Microstructure by Micro-Rs Spectroscopy

The microstructure peculiarities of the arsenoselenides were also identified with RS microscopy using the Horiba Xplora apparatus equipped with CCD detector, all micro-RS spectra being collected at ambient temperature. The CW 785 nm laser of 90 mW output power was employed for excitation, the 10% power option being used to avoid photostructural effects. Other measurement options applied were as follows: x100 objective, 1800 1/mm grating, 500 μm hole and 50 μm slit. The spectral resolution was ~2 cm^−1^, and the spatial resolution was near ~2 μm. Number of scans was chosen depending on the surface of the samples to be sure that RS spectra processed with Horiba LabSpec 6 software were reasonably identical. The milled and unmilled samples were compared through normalization by matching the spectral areas of interest. The RS active bands were identified using the known data for chalcogenide compounds [3,41,42,43,44,45,46].

### 3.4. Atomic-Deficient Microstructure by Revised PAL Analysis

The PAL spectra were recorded with the fast-fast coincidence system ORTEC (230 ps in resolution) using ^22^Na isotope of ~50 kBq activity operated in normal measuring statistics (~1 M coincidences), as described in more detail elsewhere [29]. The best fitting of the PAL spectra was achieved with the LT 9.0 program [82] under decomposition into three negative exponentials obeying normalization (*I_1_* + *I_2_* + *I_3_* = 1) and stabilizing the model-independent average positron lifetime *τ_av_*^Σ^ as the mass center of a whole PAL spectrum.

Under this measuring set-up, the reconstructed three-component PAL spectra allow for error bars in the lifetimes *τ_i_* and intensities *I_i_* at the level ±0.005 ns and 0.5%, respectively. This approach covers different channels in nanostructured substances arising from positrons annihilating in defect-free bulk states, intrinsic trapping sites (such as vacancy-type defects) and Ps-hosting holes (free-volume voids).

In nanostructured substances dominated by annihilation from positron- and Ps-related states, unconstrained three-component PAL spectra can be interpreted employing different mathematical algorithms based on canonical STM [28,29,57,83,84]. But this model is valid only for solids with one kind of trap having two-component PAL spectra. So, multichannel spectra of nanosubstances originating from a great variety of free-volume elements should be processed with STM modified for some restrictions. One of the most simplified is two-state STM ignoring Ps decaying. Within this approach, the positron-trapping modes (such as defect-specific *τ_2_* and defect-free bulk *τ_b_* lifetimes, positron trapping rate *κ_d_* and a fraction of trapped positrons *η*) can be parameterized [57,83,84]. The most plausible free-volume void modification processes in nanostructured materials can be hypothesized as suggested by Shpotyuk M. et al. [84]. Finally, the ‘remainder’ over positron trapping in the PAL spectrum is ascribed to the Ps-decay channel owing to positrons annihilating as free particles or interacting with electrons from the environment [85]. The Ps localized in holes give indication on their radii *R* in terms of the longest *τ_3_* lifetime with respect to the semiempirical Tao-Eldrup equation with material constants [85]. The fractional free volume (*f_v_*) of Ps-hosting sites can be calculated accepting the *I_3_* intensity and some constants validated for materials without structural groups inhibiting Ps formation.

Alternatively, in substances obeying three-component PAL spectra with essential input of the third component, which cannot be recompensated by source contribution, two Ps-decay scenarios can be resolved in terms of fitting parameters only [56]. The prevailing trend in the Ps formation probability would result to iso-typical changes in both *I_1_* and *I_3_* intensities accompanied by opposite changes in *I_2_* intensity. On the contrary, the prevailing trend in the hole density would result to iso-typical changes in both *I_2_* and *I_3_* intensities counterbalanced by opposite changes in *I_1_* intensity.

In heterogeneous substances obeying selective localization of traps, positron annihilation occurs through mixed positron-trapping and Ps-decaying channels. The low-electron density holes with maximum free volume and minimum surface tension should fit to confine Ps stabilization owing to its repulsive exchange potential, while regions of higher negative electron density and polarization such as sub-nm voids are more suitable to capture electrically charged positrons [86]. So, positron annihilation is expected via interconnected positron-Ps-related channels, so that Ps-decay sites are the only free-volume holes which could be converted into positron traps (and vice versa). In this case, the generalized two-state STM modified for Ps-positron trapping conversion (known as the x3-x2-CDA, Coupling Decomposition Algorithm, or the Positronics approach [28,29]) can be used. Firstly, this algorithm is applied to an unstructured substance characterized by unconstrained three-component PAL spectrum, transforming it to generalized two-component form where all trapping inputs are gathered in the second component. Then, this procedure is performed for nanostructured substances (often modified by embedded nanoparticles, NP, thus defining respective nomenclature in parameterization). Nanostructurization-driven interplay between Ps- and positron-related sites can be refined by transition to differential spectrum with the first (*τ_n_*⋅*I_n_*) and second (*τ_int_*⋅*I_int_*) components defined under condition of full inter-channel balance in nanosubstance. Parameterization of the Ps-positron trapping conversion is performed within canonical two-state STM [28,29,57,83,84] applied to this differential PAL spectrum. Finally, trapping modes such as defect-specific *τ_int_* and defect-free bulk *τ_b_^NP^* positron lifetimes, trapping rate in defects *κ_d_^NP^*, and some characteristics relevant to the size of these traps in terms of equivalent number of vacancies (*τ_int_*−*τ_b_^NP^*) and nature of these traps in terms of (*τ_int_/τ_b_^NP^*), can be determined. Within this approach, directionality in trapping conversion (Ps-to-positron or positron-to-Ps) is defined by sign of both *I_n_* and *I_int_* intensities. So, this approach can be used as indicator separating meaningful nanostructurization-driven processes from simple interplay between uncorrelated positron-trapping and Ps-decaying channels.

### 3.5. Quantum–Chemical Modeling of Molecular–Network Clustering

The optimized configurations of thioarsenide As_4_Se_n_ cage-like molecules (*n* = 6–0) and network-forming clusters derived from these molecules by breaking in available Se atom positions were reconstructed using ab initio quantum–chemical atomic cluster-modeling algorithm CINCA (Cation Interlinked Network Cluster Approach) [60,61]. The HyperChem Release 7.5 program based on restricted Hartree-Fock self-consistent field method with split-valence double-zeta basis set and single polarization function 6–311G* [87,88,89] was used. Optimization and single-point energy calculations were performed by the Fletcher–Reeves conjugate gradient method until a root-mean-square gradient of 0.1 kcal/(Å·mol) was reached. The cluster-forming energy (*E_f_*) was corrected on the energy of terminated H atoms transforming network-forming cluster into molecular one [89,90] and recalculated with respect to the energy of a single AsSe_3/2_ pyramid (*E_f_* = −72.309 kcal/mol [61]). This modelling route allows for the characterization of both molecular and network-forming conformations in covalent-bonded systems like chalcogenide glasses characterized by CN (coordination number), thus parameterizing the most energetically favorable molecular–network decomposition scenarios. To compare clusters accounting for small rings involved, the average number of constraints per atom *n_c_* was calculated following the Phillips–Thorpe constraint-counting algorithm with stretching and bending forces ascribed to intra-molecular bonds within the cluster [65,66,67].

## 4. Conclusions

Changes in the atomic-specific and atomic-deficient microstructure of binary As_x_Se_100−x_ arsenoselenides from the border of the glass-forming region (65 < *x* < 70) driven by nanomilling in dry and dry–wet modes are examined by multiexperimental characterization probes, including the X-ray powder diffraction (XRPD) in terms of the modified microcrystalline model, micro-Raman scattering (micro-RS) and revised positron annihilation lifetime (PAL) spectroscopy within the Positronics approach, complemented with molecular–network disproportionality analysis employing the ab initio quantum–chemical cluster-modeling algorithm CINCA (the Cation Interlinked Network Cluster Approach). The studied alloys are considered with respect to tetra-arsenic biselenide As_4_Se_2_ thioarsenide stoichiometry corresponding to coordination number CN = 2.67, these being glassy g-As_65_Se_35_ (CN = 2.65), glassy-crystalline g/c-As_67_Se_33_ (CN = 2.67) and g/c-As_70_Se_30_ (CN = 2.70).

Due to XRPD analysis, the crystalline inclusions in the examined alloys are ascribed to two phases enhanced under milling, especially in PVP (polyvinylpyrrolidone) water solution, such as rhombohedral As and cubic arsenolite As_2_O_3_. A straightforward interpretation of the observed changes in the amorphous structure of these alloys is developed by analyzing diffuse peak halos in their XRPD patterning recognizing the medium-range structure within the modified microcrystalline model. Disruption of the intermediate-range order due to weakening of the FSDP (the first sharp diffraction peak) responsible entities accompanied by an enhancement of the extended-range order due to fragmentation of the SSDP (the second sharp diffraction peak) responsible entities compose principal interplay between medium-range structure levels in arsenoselenides subjected to nanomilling. Since remnants of thioarsenide As_4_Se_n_ molecular entities destructed under grinding interact with oxygen, a cubic arsenolite As_2_O_3_ phase is formed in the alloys subjected to nanomilling in wet mode.

Due to micro-RS spectroscopy, the examined alloys are stabilized by thioarsenide As_4_Se_n_ molecules (preferentially with *n* = 3, 4) incorporated in the As-Se network. Nanomilling-driven molecular-to-network (amorphization) transitions occur due to the destruction of molecular thioarsenides followed by their incorporation in the network undergoing polyamorphic (reamorphization) transition. The examined arsenoselenides affected by generated defects become notably stressed, the respective defect-formation transformations being realized independently on crystalline inclusions in them.

Due to revised PAL analysis for direct conversion of bound positron–electron (Ps, positronium) states into positron traps, nanomilling-driven volumetric changes in PVP-stabilized alloys are identified with respect to dry-milled ones. Under wet milling, the Ps-hosting holes in a preferential PVP environment appear instead of positron traps with ~0.36–0.38 ns lifetimes corresponding to multiatomic vacancies in the As-Se matrix. Superposition of the PAL spectra collected for PVP, melt-quenching-derived, dry- and dry–wet-milled As-Se samples shows spectacular smoothly decaying behavior over superimposed PAL spectra peaks and tails.

The microstructure scenarios of spontaneous (under melt quenching) and activated (under nanomilling) decomposition of the As_4_Se_2_ network-forming clusters governing molecular–network disproportionality in As_x_Se_100−x_ alloys from the border of the glass-forming region (65 < *x* < 70) are recognized using the ab initio quantum–chemical cluster-modeling algorithm (CINCA). The over-constrained As_6⋅(2/3)_=As_4_ ring-like network clusters (acting as pre-cursors of rhombohedral As phase) are the main products of decomposition in As_4_Se_2_-bearing As-Se alloys. Two spontaneous processes ended by additional extraction of molecular thioarsenides with crystalline counterparts (such as dimorphite-type As_4_Se_3_ and realgar-type As_4_Se_4_) explain the location of the glass-forming border in the As-Se system near the As_4_Se_2_ composition (CN = 2.67), while the activated decomposition process ended by the extraction of layered As_2_Se_3_ network-forming entities composed of corner-shared AsSe_3/2_ pyramids is responsible for the nanomilling-driven molecular-to-network reamorphization transition in the examined alloys.

## Figures and Tables

**Table 1:** Parameterization of the FSDP-related diffuse peak halo in the examined MQ-derived arsenoselenides before and after nanomilling in dry and combined dry–wet mode.

As_x_Se_100−x_ Samples:		2*θ*	FWHM	Q_1_	ΔQ_1_	R	L	d_s_
Phase Composition, CN	State	°2*θ*	°2*θ*	Å^−1^	Å^−1^	Å	Å	Å
g-As_65_Se_35_, CN = 2.65	unmilled	15.641(5)	2.49(1)	1.11	0.18	5.66	35.52	6.96
	dry-milled	16.170(13)	4.42(4)	1.15	0.31	5.48	19.98	6.74
	dry–wet-milled	15.627(42)	6.20(12)	1.11	0.44	5.67	14.25	6.97
g/c-As_67_Se_33_, CN = 2.67	unmilled	15.479(4)	2.34(1)	1.10	0.17	5.71	37.8	7.02
	dry-milled	15.732(10)	3.77(6)	1.12	0.27	5.63	23.4	6.92
	dry–wet-milled	15.891(15)	5.08(4)	1.13	0.36	5.57	17.4	6.85
g/c-As_70_Se_30_, CN = 2.70	unmilled	15.318(5)	2.33(1)	1.09	0.17	5.78	37.90	7.11
	dry-milled	15.668(4)	4.38(5)	1.11	0.31	5.65	20.17	6.95
	dry–wet-milled	15.605(5)	8.33(12)	1.11	0.59	5.67	10.65	6.97

**Table 2:** The best-fit PAL spectra parameterization in the pelletized arsenoselenide samples in unmilled state and after nanomilling in dry- and dry–wet modes compared with these parameters in the PVP samples pelletized under the same conditions [50].

Pelletized As_x_Se_100−x_ Samples: Phase Composition, State	[FIT-1]	*τ_1_*, ns	*τ_2_*, ns	*τ_3_*, ns	*I_2_*, a.u.	*I_3_*, a.u.	*τ_av._*, ns
PVP pelletized [50]	0.200	0.196	0.472	1.867	0.256	0.119	0.466
g-As_65_Se_35_, unmilled	0.060	0.207	0.367	2.089	0.520	0.015	0.318
g-As_65_Se_35_, dry-milled	0.040	0.198	0.371	2.180	0.560	0.015	0.325
g-As_65_Se_35_, dry–wet-milled	0.070	0.216	0.431	1.937	0.390	0.046	0.379
g/c-As_67_Se_33_, unmilled	0.090	0.201	0.362	2.065	0.550	0.016	0.318
g/c-As_67_Se_33_, dry-milled	0.050	0.188	0.362	2.095	0.600	0.015	0.323
g/c-As_67_Se_33_, dry–wet-milled	0.070	0.202	0.403	1.914	0.470	0.049	0.379
g/c-As_70_Se_30_, unmilled	0.048	0.218	0.386	2.019	0.320	0.011	0.291
g/c-As_70_Se_30_, dry-milled	0.044	0.218	0.385	2.120	0.390	0.013	0.307
g/c-As_70_Se_30_, dry–wet-milled	0.070	0.212	0.425	1.730	0.329	0.062	0.377

**Table 3:** The positron trapping modes in the pelletized arsenoselenides in unmilled state and after nanomilling in dry and dry–wet modes compared with these parameters in the PVP samples pelletized under the same conditions [50], derived from their reconstructed three-component PAL spectra treated within two-state simple-trapping model (STM) ignoring Ps-decaying contribution.

Pelletized As_x_Se_100−x_ Samples: Phase Composition, State	Positron-Trapping Modes					Ps-Decay Modes	
	*τ_b_*, ns	*κ_d_*, ns^−1^	*τ_2_−τ_b_*, ns	*τ_2_/τ_b_*, a.u.	*η*, a.u.	*R_3_*, nm	*f_v_^3^*, %
PVP pelletized [50]	0.24	0.87	0.24	1.99	0.17	0.276	1.88
g-As_65_Se_35_, unmilled	0.27	1.11	0.10	1.37	0.23	0.296	0.30
g-As_65_Se_35_, dry-milled	0.27	1.35	0.10	1.38	0.27	0.304	0.32
g-As_65_Se_35_, dry–wet-milled	0.27	0.95	0.16	1.59	0.21	0.282	0.78
g/c-As_67_Se_33_, unmilled	0.27	1.22	0.09	1.35	0.24	0.293	0.30
g/c-As_67_Se_33_, dry-milled	0.27	1.56	0.10	1.36	0.29	0.297	0.30
g/c-As_67_Se_33_, dry–wet-milled	0.27	1.22	0.14	1.51	0.25	0.279	0.78
g/c-As_70_Se_30_, unmilled	0.25	0.64	0.13	1.52	0.21	0.289	0.14
g/c-As_70_Se_30_, dry-milled	0.26	0.78	0.12	1.47	0.17	0.298	0.25
g/c-As_70_Se_30_, dry–wet-milled	0.267	0.83	0.17	1.65	0.17	0.261	0.84

**Table 4:** Trapping conversion modes calculated for pelletized dry-milled arsenoselenide sample respectively to the same sample subjected to nanomilling in combined dry–wet mode.

Pelletized As_x_Se_100−x_ Sample	I Component		II Component		*τ_av_^NP^*	Trapping-Conversion Modes			
	*τ_n_*	*I_n_*	*τ_int_*	*I_int_*		*τ_b_^NP^*	*κ_d_^NP^*	*τ_int_*−*τ_b_^NP^*	*τ_int_*/*τ_b_^NP^*
	ns	a.u.	ns	a.u.	ns	ns	ns^−1^	ns	a.u.
g-As_65_Se_35_	0.193	0.315	0.363	0.433	0.286	0.261	1.37	0.09	1.35
g/c-As_67_Se_33_	0.201	0.359	0.367	0.418	0.291	0.266	1.21	0.10	1.38
g/c-As_70_Se_30_	0.220	0.477	0.376	0.321	0.283	0.264	0.76	0.11	1.43

## Data Availability

The original contributions presented in this study are included in the article. The raw data supporting the conclusions of this article will be made available by the authors on request.
